# The effect of exercise as adjunctive treatment on quality of life for individuals with alcohol use disorders: a randomized controlled trial

**DOI:** 10.1186/s12889-019-7083-8

**Published:** 2019-06-11

**Authors:** Sengül Sari, Randi Bilberg, Anette Søgaard Nielsen, Kirsten Kaya Roessler

**Affiliations:** 1 National Institute of Public Health, University of Southern Denmark, Studiestræde 6, 1455 Copenhagen, Denmark; 2 Unit of Clinical Alcohol Research, University of Southern Denmark, Campusvej 55, 5230 Odense M, Denmark; 3 Department of Psychiatry, Odense University Hospital, Winsløwsvej 20, 5000 Odense C, Denmark; 4 Department of Psychology, University of Southern Denmark, Campusvej 55, 5230 Odense M, Denmark

**Keywords:** QoL, EQ-5D, Alcohol use disorder, Exercise, RCT

## Abstract

**Background:**

A physically active lifestyle contributes to the prevention of lifestyle diseases, promotion of physical health, and reduction of pain, among other benefits. Being physically active also promotes mental health for many individuals, in the form of improved mood, increased self-efficacy and reduced risk of depression. Alcohol-dependent individuals may experience a better quality of life when supplementing their treatment with physical exercise. This study aimed to evaluate the effect of exercise on Quality of Life among patients with alcohol use disorder in a large randomized controlled trial.

**Methods:**

The study had three arms: Patients were allocated to (A) treatment as usual, (B) treatment as usual and supervised group exercise two days a week of one hour each, (C) treatment as usual and individual physical exercise minimum two days a week. Duration of the intervention was six months. Data on values of Quality of Life were collected at baseline (before treatment start and at time of enrollment in the study), and at follow-up (at six months after enrollment in the study) using the EQ-5D questionnaire and the EQ-VAS. The sample consisted of 117 consecutive patients, and the follow-up rate was 66.6%. Intention-to-treat analyses were conducted to evaluate the effect of exercise on quality of life.

**Results:**

Although not statistically significant, a substantial portion of the participants in the individual exercise condition reported that they had no pain or discomfort (one of the five quality of life dimensions measured by EQ-5D questionnaire) compared to the controls at follow-up. No difference was found between the groups regarding the EQ-VAS.

**Conclusion:**

The exercise intervention had no effect on quality of life for patients with alcohol use disorder, nor was quality of life improved across the total sample. More research in how to improve quality of life for patients with alcohol use disorder is needed.

**Trial registration:**

ISRCTN74889852 (retrospectively registered, date: 16/05/2013).

## Background

Alcohol use disorder (AUD) is a preventable and treatable disorder, yet the prevalence of alcohol dependency and hazardous use is high in the Danish population [1]. Moreover, it is estimated that only 10 % of alcohol-dependent individuals receive treatment [2], even though treatment is publicly financed and free at the point of use. Further, treatment of AUD is associated with a range of compliance factors, which means that even when an individual enters the treatment facility, his/her risk of treatment interruption and relapse remains high [3]. Research in the AUD treatment area suggests several interventions to support adherence to treatment and better treatment outcome. Particularly in the later stages of treatment, focusing on reintegration into society and restoration of normal functioning, including the adoption of a healthy lifestyle has been found important [4]. One aspect of a healthy lifestyle is physical activity [5, 6]. Interventions involving physical exercise as an adjunct to treatment for alcohol use disorder are therefore suggested to have promising effects [7–11].

Previous studies of the use of exercise in AUD treatment have shown positive effects on treatment outcome; both in regard to alcohol-specific outcomes, such as intake, craving and withdrawal symptoms, and in regard to other health-related outcomes, such as mood, cognitive function and quality of life [8, 12]. Quality of life has been shown to be significantly impaired in those with alcohol abuse and dependence [13–16], particularly in the domains of mental health and social functioning [17]. But although favorable effects of exercise on quality of life were found, the evidence for the efficacy of exercise in alcohol and drug treatment is weak [7]. Methodological limitations, such as too small sample sizes, non-generalizable populations and the lack of intention-to-treat-analyses to correct for the high number of dropouts were some of the factors limiting the strength of the evidence [7]. Preliminary evidence for the role of exercise as an adjunctive tool in the treatment of AUDs and substance use disorders (SUDs) is, however, promising [18, 19].

Recently, two feasibility studies of exercise as an adjunctive treatment for AUD and SUD, respectively, showed significant improvements in both physical activity levels and quality of life for the participants [12, 20]. Regular exercise was also found to be a significant predictor of health-related quality of life (HRQoL) and substance use dependence [21], and exercise, along with other health-related behaviors, was found to be strongly associated with HRQoL among veterans in addictions treatment [22]. Bearing in mind that the evidence is still somewhat limited, exercise appears to be an alternative and effective substance-free activity and a relapse prevention strategy in the treatment of both AUDs and SUDs [23, 24]. Consequently, implementing exercise into the existing treatment of AUDs and SUDs may be a promising strategy for improving the quality of life of participants, which may in turn support the outcome of treatment on other parameters. However, well-designed and carefully performed randomized controlled trials of exercise as an adjunct to treatment of AUD are needed. Furthermore, information on how exercise may affect secondary outcomes of the treatment, such as patients’ quality of life, is not sufficiently covered in the available literature so far.

Several theoretical and practical factors provide further support for exercise-based treatments for AUDs and SUDs, including the psychological, behavioral, and overall positive health effects [18]. The psychological mechanisms of exercise on individuals with SUDs include, for example, the reduction of cravings and withdrawal symptoms, and may thus prevent relapse both acute and long term [25–27]. An example of the effect of the behavioral mechanisms of exercise for alcohol- or other substance-dependent individuals is that when urges arise, engaging in an alternative behavior such as physical activity instead of drinking or using drugs may help reduce relapse [18]. Furthermore, psychobiological research has demonstrated that both exercise and commonly abused substances activate the same reward areas in the brain [28, 29]. This so far small but growing body of research supports the relevance of using exercise as a treatment for substance use disorder. Theoretically, exercise programs can be lifestyle interventions that provide people with AUDs with skills to undertake a positive health promoting behavior, in addition to simultaneously provide self-control strategies, coping strategies and an alternative to drinking [30, 31]. The concept Runner’s high, which is the feel-good effect of running due to release of endorphins that has been considered as natural painkillers [32], may also have potential to substitute alcohol with exercise [33]. Therefore, randomized controlled trials with appropriate sample sizes, which are also more generalizable to populations with alcohol dependence, are needed to evaluate the effect of exercise-based interventions on alcohol use and related outcomes.

The Healthy Lifestyle Study, conducted in a Danish outpatient alcohol treatment setting, investigated the effect of physical exercise on alcohol use disorder [34–36]. Data were collected on primary outcomes, in terms of alcohol consumption, amount and frequency, and secondary outcomes, including quality of life, depression, and interpersonal problems were investigated. Regarding the primary outcomes, no direct effect of physical exercise on alcohol consumption was found, probably due to low adherence to the exercise intervention. However, supplementary findings of the study were quite promising. The intervention effect on primary outcome which was *consumed amount of alcohol per week* showed an OR of 0.99 [95% CI: 0.46; 2.14], *p* = 0.976 for excessive drinking in the group exercise condition, and 1.02 [95% CI: 0.47; 2.18], *p* = 0.968 in the individual exercise condition, which, when compared to the control group as reference, did not differ statistically significantly. This may indicate that the AUDs treatment itself is successful regardless of the intervention. However, participants with moderate level general physical activity had lower odds for excessive drinking OR = 0.12 [0.05; 0.31], *p* < 0.001 than participants with low level general physical activity. Furthermore, the amount of alcohol consumption in the intervention groups decreased by 4% [95% CI: 0.03; 6.8], *p* = 0.015 for each increased exercising day. This suggests a dose-response effect of exercise on drinking outcome and supports the need for implementing physically active lifestyles for patients in treatment for alcohol use disorder [34].

This paper aims to evaluate the effect of the intervention on quality of life for subjects who participated in the Healthy Lifestyle Study.

## Methods

### Design and interventions

This article demonstrates findings on secondary outcomes of a randomized controlled trial called the Healthy Lifestyle Study. Therefore, the methodology is described in detail in a previously published article [34]. In brief, the intervention was adjunct to treatment as usual consisting of running and brisk walking for consecutive patients receiving outpatient treatment for alcohol use disorders, randomly allocated to either supervised group training or individual self-organized training. They were asked to complete a 24-week program, either alone (*N* = 40) or in a training group (*N* = 41). Participants in the individual (self-organized) exercise group received an individual program and running instructions during two individual sessions prior to start, following which they were asked to organize their exercise sessions themselves. The duration of the exercise activity described in the training program began with 15 min in the first week, gradually increasing to 60 min in the final weeks. Participants in the supervised exercise group were asked to meet with their group and instructors twice a week for one-hour training sessions. Exercise activity was recorded using a heart-rate monitor (Polar RC3 GPS with Heart Rate Sensor). The control group received treatment as usual. Randomization was not blinded for assessors. The study adheres to CONSORT guidelines [34, 35].

### Setting

The study was conducted in an alcohol outpatient treatment center in Denmark, where the municipalities are legally responsible for the provision of treatment for alcohol use disorders free of charge. The treatment offered consists of motivational interviewing, cognitive behavioral therapy and family therapy. Further, acute treatment for withdrawal symptoms and other kinds of pharmacological treatment may also be offered. Anonymity during treatment is optional [37].

### Participants

A total of 175 consecutive participants were recruited at the time of the initiation of their treatment course for AUDs and met the following inclusion criteria during May 2013 to March 2015: fulfilling ICD-10 criteria for harmful use of or dependence on alcohol, aged over 18 years, Danish-speaking, no severe psychosis or cognitive impairment, no severe physical disabilities or medical problems, and acceptance of participation in the study. All participants provided written and oral informed consent. Of the 175 participants, 58 failed to return the baseline questionnaires (dropouts from study and/or dropouts from treatment), which led to a final sample of 117 participants for this present investigation. Thus, this study is based on a subpopulation of the total sample. Of these, 36 were randomized to the control group, 41 to the group exercise condition, and 40 to the individual exercise condition. The completers of the baseline questionnaire did not differ significantly from those who failed to complete it, except for the alcohol composite score of the Addiction Severity Index (Table 1), where non-completers had a higher score than completers.

**Table 1:** Participant demographics and clinical variables with comparisons between completers and non-completers, and baseline information from EQ-5D and EQ-VAS from baseline among completers

Variable name	Non-completers (*N* = 58)	Completers (*N* = 117)	*p*-value
Age mean (SD)	45.9 (10.6)	44.6 (11.6)	0.461
Male N (%)	38 (65.5)	84 (71.8)	0.395
Education > 9 yrs. N (%)	37 (63.8)	87 (74.4)	0.148
Employed N (%)	32 (55.2)	66 (56.4)	0.877
Cohabiting N (%)	16 (27.6)	43 (36.8)	0.227
Alcohol consumption median (IQR)^a^	237 (102;400)	201 (72;361)	0.162
Alcohol Composite Score median (IQR)^b^	0.80 (0.63;0.90)	0.71 (0.50;0.85)	0.023
EQ-VAS median (IQR)		70 (50;80)	
*EQ-5D*	*Degree of disability*	*N (%)*	
*Mobility*	*None*	*100 (85.47)*	
	*Moderate*	*15 (12.82)*	
	*Severe*	*0*	
*Self-care*	*None*	*113 (96.58)*	
	*Moderate*	*2 (1.71)*	
	*Severe*	*0*	
*Usual activities*	*None*	*76 (64.96)*	
	*Moderate*	*33 (28.21)*	
	*Severe*	*5 (4.27)*	
*Pain/discomfort*	*None*	*57 (48.72)*	
	*Moderate*	*51 (43.59)*	
	*Severe*	*7 (5.98)*	
*Anxiety/depression*	*None*	*61 (52.14)*	
	*Moderate*	*47 (40.17)*	
	*Severe*	*5 (4.27)*	

^a^Total number of standard units of alcohol consumed in the 30 days prior to treatment initiation (TLFB)

^b^Calculated by means of the Addiction Severity Index (ASI), 0 (no problem) to 1 (severe problem)

## Measures

### Quality of life

The EuroQOL five dimensions questionnaire (EQ-5D) [38, 39] is one of the most commonly used generic questionnaires to measure health-related quality of life (HRQOL). The conceptual basis of the EQ-5D is a holistic view of health, which includes the medical definition, as well as the fundamental importance of independent physical, emotional and social functioning. The concept of health in EQ-5D also involves both positive aspects (well-being) and negative aspects (illness). The EQ-5D consists of a questionnaire and a visual analog scale (EQ-VAS). The EQ-VAS is a self-rated health status scale using a VAS, which records the subject’s perceptions of their own current overall health and can be used to monitor changes with time. The self-assessment questionnaire is a self-reported description of the subject’s current health in five dimensions: mobility, self-care, usual activities, pain/discomfort and anxiety/depression. The subject is asked to grade their own current level of function in each dimension into one of three degrees of disability: severe, moderate or none.

### Addiction severity

The Addiction Severity Index (ASI) [40] provides a multidimensional image of the patient’s psychosocial and addiction situation within the last month before the interview. The interview concentrates on the following seven areas in the patient’s life: medical status, employment, alcohol use, drug use, legal status, family or social network, and psychiatric health. ASI provides two different scores: the interviewer score and the composite score. The scores give an estimate of each problem area based on symptoms within the 30-day period preceding the interview. Each composite score consists of the sum of the various items from the ASI. Final scores are reported as 0 to 1, where 0 denotes no problems and 1 denotes severe problems.

## Analysis

A comparison analysis of demographic variables and baseline alcohol variables was conducted to determine whether there was a difference in the study sample between those participants who completed all the baseline questionnaires (completers) compared to those who failed to complete them all (non-completers). Chi-square tests, t-tests and non-parametric Wilcoxon Signed-Rank tests were used where appropriate to examine for significant (*p* = 0.05) differences.

In 39 (33.3%) cases, follow-up information was missing. Last observation carried forward (LOCF) was conducted to address the problem of missing data in an intention-to-treat analysis.

Differences between the control and intervention groups on the five quality of life dimensions were investigated using Fisher’s exact test to analyze the distribution of participants in each degree of disability.

A Wilcoxon signed-rank test was used to compare follow-up and baseline EQ-VAS for the total sample. Analysis of covariance (ANCOVA) controlling for baseline EQ-VAS was utilized to evaluate differences of follow-up EQ-VAS between groups. Level of statistical significance was determined at *p* < 0.05 for all analyses.

## Results

Baseline demographic and clinical characteristics of the sample are presented in Table 1. No statistically significant differences in demographic and clinical characteristics between the completers and non-completers of the EQ-5D and EQ-VAS questionnaires were observed, except for the alcohol composite score calculated using the ASI. The non-completers had higher alcohol composite scores on average, which means that they had more severe alcohol problems than the completers.

**Fig. 1:**
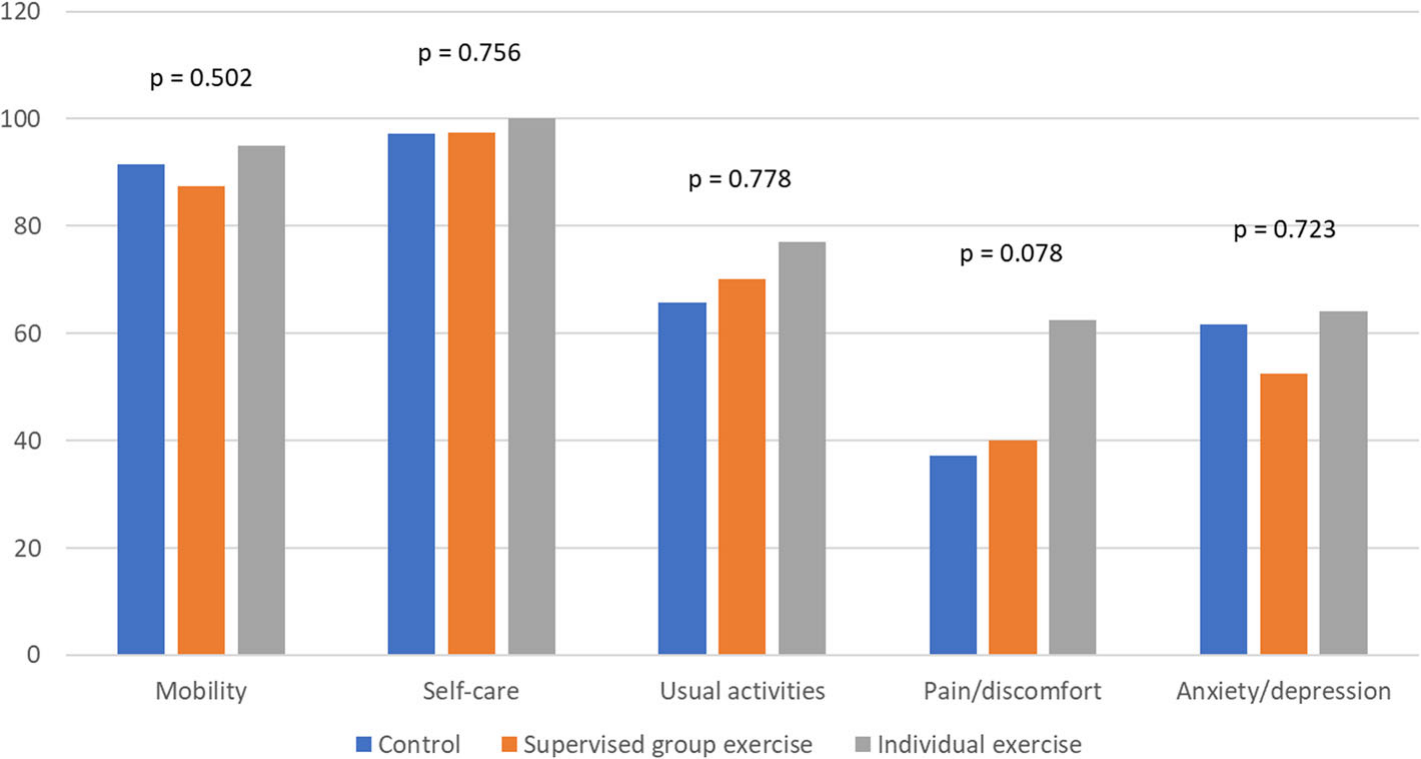
Percentage of participants in each group who rate their degree of disability as “none” in each dimension of the EQ-5D at follow-up, 6 months after treatment start, intention to treat, *N* = 117, *p* = Fisher’s exact test for difference between groups in all degrees of disability

The intention to treat analyses of intervention effect on quality of life showed no statistically significant difference between intervention groups in EQ-5D at follow-up. Figure 1 illustrates the frequency of participants in each group rating their level of function in each dimension and assessing their degree of disability as “none”, 6 months after treatment start and thereby end of intervention. Here 62% of the participants in the individual exercise condition reported that they had no pain or discomfort compared to 38% of the controls reporting the same (*p* = 0.078). This indicates a trend towards an exercise intervention effect on pain and discomfort for patients in treatment for AUD.

Self-rated health status, as assessed by the EQ-VAS, did not differ at a statistically significant level between intervention groups at follow-up as analyzed by ANCOVA (Table 2). Neither did the change in mean EQ-VAS from baseline to follow-up across the total sample.

**Table 2:** Participants’ self-rated health status on the EQ-VAS at 6 months after treatment start, LOCF

EQ-VAS *N* = 117	Median (IQR)	*p*-value
Control	69.5 (58;80)	
Supervised group	70 (50;80)	
Individual exercise	72.5 (68.5;83.5)	
Difference between groups		0.1540*
Total	70 (55;80)	0.8493**

*ANCOVA controlling for baseline EQ-VAS, **Wilcoxon signed-rank test, compared with baseline EQ-VAS for the total sample

## Discussion

Similarly to the primary outcome analysis [34], the present study found no significant differences in quality of life dimensions between the groups participating in the exercise intervention and the controls. Quality of life for individuals with alcohol use disorders is generally suggested to improve during treatment, as patients may experience better control and fewer problems in everyday life while staying abstinent or drinking sensibly. Moreover, the very fact of measuring and monitoring QoL itself during assessment and treatment may add important value to patient recovery [17]. However, no significant change in the present study was observed. It is also suggested that alcohol-dependent individuals experience improvements in QoL across treatment with both short-term and long-term abstinence, and even in the absence of complete abstinence, a marked reduction in drinking is associated with significant increases in QoL [13].

Results of the present study can therefore not support earlier findings in that that individuals with alcohol use disorders included in this study did not increase their quality of life. Unlike earlier studies, our study had a longer follow-up period, but we found no effect of the intervention on primary outcome at the six months follow-up, which supports the findings of Brown et al. [41]. This means that the potential effect of adding exercise interventions to treatment as usual may dissolve on long term. Probably participants’ motivation and adherence to exercise declines in the months after initiation, maybe due to the lack of social support and individually tailored exercise programs, which we found were important reasons to drop out [42], and this may explain why no intervention effect was observed at six months follow-up.

Furthermore, the dropout rate in our study was 37.1%, which is a little lower than the 40% reported in a recent meta-analysis by Hallgren et al. [8]. However, a slight tendency towards better outcome among participants allocated to individual exercise is seen, especially in the pain dimension. A large body of research on physical exercise for pain management suggests that exercise has a pain-reducing effect [43–46]. Therefore, although not significant, a substantial portion of the participants in the individual exercise condition reporting that they have no pain or discomfort compared to the controls is interesting, as this finding supports the suggested pain reducing effect of exercise demonstrated in prior research. As suggested by Roessler [47], physical exercise can provide important support in the treatment of drug abuse. In terms of quality of life dimensions, pain is an important dimension which should be further studied for this particular target group.

In addition, it is not surprising that there was no difference between the groups in the mobility and self-care dimensions. This is mainly because the study was conducted in an outpatient treatment center, where patients in general are expected to have a relatively high degree of independency and high level of physical function [48]. If participants were inpatients, the picture would probably be different.

Regarding the effect of exercise on quality of life for the study participants, it is interesting that no significant differences between intervention and control groups were observed at follow-up. This may be due to a relatively high number of participants not exercising as much as expected, as discussed elsewhere in relation to the primary outcomes of the study [34]. It may also partially be explained by a relatively high dropout rate from the study, since one third of the included participants did not return the follow-up questionnaire [43]. Consequently, baseline values of quality of life were used to determine follow-up values for one third of the study sample. This may have affected the analyses and results conservatively, thus explaining why we could not observe any improvement in quality of life.

### Limitations

As mentioned above, a very important study limitation was the relatively high number of dropouts (33.3%), thus leaving the study unable to conduct measurements across the whole sample at follow-up. In particular, since we have no information about the quality of life at follow up on the non-completers, and since the non-completers presented higher alcohol severity composite scores at baseline, our findings may not cover patients with more severe alcohol addiction. In addition, it is possible that the type or level of activity in the intervention was insufficient to induce change. Furthermore, the EQ-5D used for measuring quality of life in this study may lack the sensitivity to measure change over time [49, 50], and with some patient or population groups it has shown mixed validity [51]. Moreover, EQ-5D, chosen for use in this study mostly for its user-friendly character, is a short questionnaire [52]. Finally, the power calculation for the present study was made on the primary outcome variable. In addition, it proved difficult to recruit the estimated number of participants. It is thus a limitation of the present sub-study that no power calculation was made to estimate the number of included patients necessary to detect differences in relation to changes in quality of life, and we cannot rule out that the present sub-study is under-powered.

### Strengths

In this study, all the participants were consecutive patients and recruited from a real treatment institution, which means that it was not a study of extraordinarily motivated patients. This enabled the study to test the effectiveness of the intervention in an actual setting and describe realistic findings.

## Conclusion

No improvements in quality of life were observed when adding physical exercise to treatment of alcohol use disorder, but a trend towards reduction in pain and discomfort among participants who exercised individually was observed. Future research will seek to further investigate and advance the quality of life for individuals with alcohol use disorder and make suggestions of how to improve the quality of life for this population group.

## Data Availability

Data is owned by primary investigator at the Healthy Lifestyle Study, together with the research group behind the RESCueH-studies and stored safely. Collection of the data is approved by the Danish Data Protection Agency (https://www.datatilsynet.dk/english/legislation/). According to the Danish Data Protection Agency (which regulates personal data access in Denmark) it is not possible to publicly deposit the data, as this will violate the requirements of the Danish Act on Processing of Personal Data of 2002 (amended in 2012). Data will instead be available through Danish National Archives, which is the official Danish platform for safe data storage. Interested researchers can apply for access to the data by following this link https://www.sa.dk/en/services/the-research-service-of-the-danish-national-archives/

## Abbreviations

ANCOVA: Analysis of covariance
ASI: Addiction Severity Index
AUD: Alcohol use disorder
EQ-5D: EuroQOL five dimensions questionnaire
HRQoL: Health-related quality of life
ICD-10: International Classification of Diseases and Related Health Problems 10th version
IQR: Inter quartile ranges
LOCF: Last observation carried forward
QoL: Quality of life
RESCueH: Acronym for the five randomized controlled trials of interventions in treatment of alcohol use disorder: the Relay Study, the Elderly Study, the Self-match Study, the Cue Exposure study and the Healthy Lifestyle Study
SUD: Substance use disorder
TAU: Treatment as usual
TLFB: Time-line Follow Back
VAS: Visual analog scale
